# Bone Morphogenetic Protein Antagonist Gremlin-1 Increases Myofibroblast Transition in Dermal Fibroblasts: Implications for Systemic Sclerosis

**DOI:** 10.3389/fcell.2021.681061

**Published:** 2021-06-04

**Authors:** Laura Duffy, John Henderson, Max Brown, Stefan Pryzborski, Nicola Fullard, Lena Summa, Jorg H. W. Distler, Richard Stratton, Steven O’Reilly

**Affiliations:** ^1^Faculty of Health and Life Science, Northumbria University, Newcastle upon Tyne, United Kingdom; ^2^Biosciences Department, Durham University, Durham, United Kingdom; ^3^Department of Internal Medicine 3 Friedrich-Alexander-University, Erlangen-Nurnberg, Germany; ^4^Centre for Rheumatology, University College London, London, United Kingdom

**Keywords:** fibrosis, ECM, BMP, gremlin-1, collagen

## Abstract

**Objective:**

Systemic Sclerosis is an autoimmune connective tissue disease which results in fibrosis of the skin and lungs. The disease is characterized by activation of myofibroblasts but what governs this is unknown. Gremlin-1 is a BMP antagonist that is developmentally regulated and we sought to investigate its role in Systemic Sclerosis.

**Methods:**

Dermal fibroblasts were transfected with Grem1pcDNA3.1 expression vectors or empty vectors. Various markers of myofibroblasts were measured at the mRNA and protein levels. Scratch wound assays were also performed. Media Transfer experiments were performed to evaluate cytokine like effects. Various inhibitors of TGF-β signaling and MAPK signaling were used post-transfection. siRNA to Gremlin-1 in SSc dermal fibroblasts were performed to evaluate the role of Gremlin-1. Different cytokines were incubated with fibroblasts and Gremlin-1 measured. Bleomycin was used as model of fibrosis and immunohistochemistry performed.

**Results:**

Overexpression of Gremlin-1 was achieved in primary dermal fibroblasts and lead to activation of quiescent cells to myofibroblasts indicated by collagen and α-Smooth muscle actin. Overexpression also led to functional effects. This was associated with increased TGF-β1 levels and SBE luciferase activity but not increased Thrombospondin-1 expression. Inhibition of Gremlin-1 overexpression cells with antibodies to TGF-β1 but not isotype controls led to reduced collagen and various TGF-β pathway chemical inhibitors also led to reduced collagen levels. In SSc cells siRNA mediated reduction of Gremlin-1 reduced collagen expression and CTGF gene and protein levels in these cells. IL-13 did not lead to elevated Gremlin-1 expression nor did IL-11. Gremlin-1 was elevated in an animal model of fibrosis compared to NaCl-treated mice.

**Conclusion:**

Gremlin-1 is a key regulator of myofibroblast transition leading to enhanced ECM deposition. Strategies that block Gremlin-1 maybe a possible therapeutic target in fibrotic diseases such as SSc.

## Introduction

Gremlin-1 is a highly conserved known bone morphogenetic protein (BMP) antagonist which is critical in embryogenesis (Brazil et al., 2015). It is a member of the Transforming Growth Factor-β superfamily and includes Gremlin-1 and noggin (Stafford et al., 2011; Brazil et al., 2015). BMPs are glycosylated ligands that induce bone formation and as well as that can regulate development of adipose, neurological, ophthalmic, cardiovascular, and musculoskeletal systems and as such are morphogens (Schmidt et al., 1995; Nickel and Mueller, 2019). BMPs usually function by short range diffusion and bind serine/threonine kinase receptors (type I and type II receptors), which triggers phosphorylation of receptor induced Smads (Nickel and Mueller, 2019). The main Smads activated downstream of BMP receptors are R-Smad 1/5/9. BMP signaling is tightly regulated by multiple extracellular binding proteins which regulate BMP diffusion and thus control BMP signaling spatially and temporally. Gremlin-1 is the major BMP antagonist that maintains proper limb development during embryogenesis and aberrant expression in adults is associated with orofacial cleft (Mangold et al., 2010) and various cancers (Jang et al., 2017; Lieberman et al., 2017).

Systemic Sclerosis (SSc) is an idiopathic autoimmune disease characterized by inflammation, vascular abnormalities and fibrosis (Denton and Khanna, 2017; Hinchcliff and O’Reilly, 2020). The etiology of the disease remains unknown and few treatments are available. The disease is mainly associated with skin and lung fibrosis with increased extracellular matrix, in particular, collagen, underlying the pathology of the disease (Henderson et al., 2019b). Activated myofibroblasts are key cell types mediating the deposition of ECM leading to fibrosis but what precisely governs their activation remains elusive (Kisseleva and Brenner, 2008; Volkmann and Varga, 2019). Given that development pathways reactivation is associated with cancer it could be proposed that such pathways are operative in SSc (Bertrand et al., 2012).

Indeed, one of the hallmarks of cancer is reactivation of the pathways that control cellular differentiation during development and in relation to SSc, aberrant activation of Wnt pathways drive fibrosis. Increased levels of the BMP antagonist Gremlin-1 are associated with fibrosis of the kidney (Dolan et al., 2005; Li et al., 2012),liver (Yang et al., 2012), lung (Koli et al., 2006), and ocular system (Lee et al., 2007). We have also previously identified Gremlin-1 as mediating fibrosis in dermal fibroblasts after Interleukin-6 stimulation and elevated levels have been demonstrated in PCR arrays from SSc tissue biopsies (Meyringer et al., 2007). This study aims to evaluate the role of the BMP antagonist Gremlin-1 is SSc and to determine its signaling pathway(s).

## Materials and Methods

### Cell Culture

Dermal fibroblasts were obtained from 4 healthy control derived from skin tissue from female healthy donors undergoing surgery for adipose tissue removal. Cells were maintained in DMEM with the addition of 10% (v/v) heat inactivated fetal calf serum (Gibco), 100 units/ml penicillin (Life technologies, United Kingdom) and 100 μg/ml streptomycin in 5% CO_2_ at 37°C in an incubator until required for experimental protocols. SSc dermal fibroblasts were taken from 3 patients that have been clinically diagnosed with early diffuse SSc by a clinical consultant rheumatologist. Early being defined as <2 years from the first non-Raynaud’s symptom. All three patients were female and were treatment naïve. Local research ethics was obtained for this from the NHS NRES committee, Hamsted, London reference 6398. Lesional skin biopsies were taken by punch biopsy and cultured in standard media described above until the fibroblasts had outgrown and then the cells were removed pelleted and washed three times and replated into standard tissue culture plates.

### pcDNA3.1a Plasmid Generation and Transfection

A DNA fragment containing the entire coding sequence for Gremlin-1 was amplified by RT-PCR on cDNA from HK-2 cells using forward primer 5′- GACAGTGAATTCA TGAGCCGCACAGCCTACACG-3′ and reverse primer 3′- GGATTTTCTAGAATCCAAATCGATGGATATGCA-5′ and RedTaq^®^ DNA polymerase at an annealing temperature of 55°C and was ligated into pcDNA3.1/myc-his (Invitrogen) and transformed into competent cells as described (Church et al., 2015). Positive colonies from LB plates were picked and grown in LB supplemented with selection antibiotic and plasmids isolated using maxiprep kits (Qiagen, United Kingdom).

Actively dividing dermal fibroblasts were trypinised and 75,000 cells seeded into an Eppendorff and pelleted. The cell pellet was then resuspended in Ingenio^®^ solution (Mirus bio, Wisconsin, United States) and transferred to a 4 mm electroporation cuvette (Cell Projects Ltd., Kent, United Kingdom) with 10 μg of corresponding pDNA or pDNA combined with 80 nM siRNA, Scramble—5′AATTCTCCGAACGTGTCACGT3′ Gremlin-1–5′CTGCCGGCTGCTGAAGGGAAA3′. A single electrical pulse of 960 μF, 250 V and Ohms was delivered using a Bio-Rad Gene Pulser. Cell debris removed after 4 h and viable cells incubated for the appropriate time course.

### Western Blotting

Cell lysates were obtained using RIPA buffer and the protein quantified using a BCA assay (Thermo Fisher Scientific, Massachusetts, United States) 10 μg of total protein was ran on a denaturing SDS-PAGE and transferred via wet transfer to 0.45 μM Nitrocellulose paper. The blot was then blocked using 5% milk PBST and incubated overnight at 4°C in either Collagen 1 1:500, (Abcam, ab138492), Myc 1:1,000, (Cell Signaling technologies, Danvers, United States #2276), alpha SMA 1:1,000, (Abcam, ab7817), and Alpha tubulin 1:2,000, (Abcam, ab7291) used a loading control. The membrane was then washed, and secondary antibody conjugated to Horseradish peroxidase (HRP) (Sigma Aldrich, United Kingdom) applied at room temperature for 1 h. Blots were visualized using ECL Prime (GE Healthcare, Illinois, United States) and G:box (Syngene, United States).

### IL-6 and IL-11 Stimulation Experiments

Dermal fibroblast was seeded into 6 well tissue culture plates to adhere they were then washed twice with PBS and stimulated with 20 ng/ml recombinant IL-6 and 25 ng/ml recombinant soluble IL-6 receptor (R&D systems, United Kingdom). They were then incubated for 48 h in culture after which the cells were lysed and qRT-PCR was performed for Gremlin-1 as described. In some experiments the IL-6 and soluble IL-6 stimulation was pretreated for 1 h with the STAT3 inhibitor S31-201 (Sigma Aldrich, United Kingdom) 15 μM in DMSO, which was previously demonstrated to be antifibrotic in SSc dermal fibroblasts (Chakraborty et al., 2017). After the appropriate time the cells were lysed and Gremlin-1 quantified by qRT-PCR.

In some experiments the IL-6 was replaced with human recombinant IL-11 (R&D systems, United Kingdom) at 10 ng/ml. This dose was chosen based on dose response performed in our recent publication in which we performed dose response curves (Adami et al., 2021).

### IL-13 Stimulation Experiments

Healthy dermal fibroblasts were culture in standard six well plates until confluent. Media was removed and replaced with media containing recombinant IL-13 100 ng/ml (R&D Systems, United Kingdom) at which we have previously demonstrated to give a robust increase in collagen at both mRNA and protein levels in fibroblasts (O’Reilly et al., 2016). After 24 and 48 h cells were lysed and Gremlin-1 quantified by qRT-PCR. At 24 h Col1A1 was also quantified by qRT-PCR.

### Quantitative RT-PCR

RNA extraction was performed as per the Qiagen RNeasy kit (Qiagen, Hilden Germany) and 1 μg converted to cDNA using Nanoscript 2 reverse transcriptase (Primer Design Ltd., Southampton, United Kingdom). A master made up of 100 nM of forward and reverse primers (Table 1), precisionFAST qPCR Master Mix (Primer Design Ltd., Southampton, United Kingdom) and 5 μl cDNA. Relative levels were normalized to 18s and compared using the ΔΔCt method to obtain fold change relative to control as previously described (Henderson et al., 2019a).

**TABLE 1:** PCR primer sequences used in this study.

**Gene**	**Forward primer**	**Reverse primer**
GREM1	5′ TAT GAG CCG CAC AGC CTA CA-3′	5′ GCA CCT TGG GAC CCT TTC TT 3′
COL1A1	5′ CAA GAG GAA GGC CAA GTC GAG G 3′	5′ CGT TGT CGC AGA CGC AGA T 3′
CTGF	5′ AAT GCT GCG AGG AGT GGG T 3′	5′CGG CTC TAA TCA TAG TTG GGT CT 3′
18s	5′ CGA ATG GCT CAT TAA ATC AGT TAT GG 3′	5′ TAT TAG CTC TAG AAT TAC CAC AGT TAT CC 3′

### Media Transfer

Post-transfection cells were incubated for 48 h. After this time conditioned media was then removed and placed on to un-transfected healthy dermal fibroblasts different from those transfected. This media was then left for a further period of time of 48 h after which cells were lysed and subjected to Western blotting with specific antibodies.

### Scratch Assay

Post electroporation confluent healthy dermal fibroblasts were in 24 well plates at 100,000 cells/well, then left to adhere in standard DMEM media as described. After 24 h they were scratched with a p5 sterile pipette tip and images taken using a Leica DMi1 microscope every 8 h, analysis of this images conducted via Image J software and% wound closure obtained by deriving from the wound area.

### Small Interfering RNA Transfection of Gremlin-1

SSc dermal fibroblasts from 3 independent early (<2 years from non-Raynauds symptom) diffuse SSc donors were seeded into 24 well plates and transfected with siRNA (80 nM) scramble or Gremlin-1 specific siRNA using DharrmaFECT1© transfection reagent (Thermo, United Kingdom) according to the manufacturers instructions. Forty-eight hours post-transfection of either scramble or Gremlin-1 siRNA cells were lysed and RNA harvested to confirm Gremlin-1 knockdown at the mRNA levels using qPCR. Data was normalized to the housekeeping gene 18S. In some experiments cells were lysed and subjected to Western blotting for CTGF protein expression 1:300, (Ab6992) (Abcam, United Kingdom).

### Enzyme Linked Immunosorbent Assay (ELISA)

After transfection media was collected and stored immediately at −80°C until used further. Media was measured for TGF-β1 and Thrombospondin-1 (TSP-1) by standard specific ELISA (R&D Systems, United Kingdom) following the manufacturer’s instructions. All samples were run in triplicate with internal controls.

### SBE Luciferase Assay

Plasmids containing 4 repetitions of GTCTAGAC Smad3/4 binding motif element with luciferase was gifted by Prof. Jorg Distler. This was transfected into healthy dermal fibroblasts using lipofectamine 2000 (Invitrogen, United Kingdom) per manufacturer’s instructions at 1.1 μg and 6 h post transfection cells were washed twice in warm PBS and media was added from empty plasmid or Gremlin-1 transfected fibroblasts and then left for 6 h after which time luciferase was measured using a luminometer. Data is expressed as percentage compared to empty pcDNA3.1 vector media only set at 100%. In some experiments recombinant TGF-β1 was added (10 ng/ml) (R&D systems, United Kingdom) plus and minus neutralizing TGF-1 antibody 1 μg/ml (Mab1835 clone; R&D systems, United Kingdom) to confirm specificity of the antibody in use.

### Sircol Assay

After specific experimental conditions explained the media was removed from the cells and analyzed by the Sircol Assay (Biocolor, Newtontown abbey). Briefly media was removed and centrifuged at 377 g for exactly 5 min. One milliliter of Sircol dye was added to 200 μl of supernatant and incubated for 30 min after which the complex was centrifuged at 16,770 g for 10 min to spin the formed collagen-dye complex. After removal of the supernatant droplets were dissolved in 1 ml Sircol alkali reagent and absorbance measured at 540 nm on a spectrophotometer. A standard curve was constructed and data was read of the standard curve.

### TGF-β Inhibitor Treatment Experiments

Immediately post transfection with pcDNA3.1 or pcDNA3.1Grem1, cells were treated with antibodies to TGF-β1 at 1 μg/ml (Mab1835 clone; R&D systems, United Kingdom) or a matched isotype control antibody (1 μg/ml) in 24 well plates to neutralize TGF-β1 levels in the media of the cells. After 48h the media was measured for collagen by Sicrol assay as described above. As described above cells were transfected with SBE luciferase plasmid plus and minus TGF-β1 and neutralizing antibody. To inhibit the TGF-β receptor post transfection we immediately treated the cells with LY2109761 (Sigma Aldrich, United Kingdom) at 70 nM and post 48 h the media was collected and collagen quantified. To determine the role of the TGF-β non-canonical pathway we incubated the dermal fibroblasts with U0126, an MEK inhibitor (Cell Signaling, United Kingdom) 10 μM in DMSO, transfection or not and after 48 h the cell media was harvested and collagen measured by the Sircol assay.

### MTT Assay

SSc dermal fibroblasts were cultured in 96 well plate and transfected with either scramble siRNA or Gremlin-1 specific siRNA (80 nM) using DharmaFECT1© (Thermo, United Kingdom) according to manufacturers instructions, 48 h post transfection cells were subject to the MTT assay (Roche) which is based on the cleavage of tetrazolium salt. The plate was read in a plate reader at 570 nm. Data is presented at absorbance at 570 nM from *n* = 3 SSc donors.

### Gremlin-1 Immunohistochemistry

Mice were treated with either NaCl or injections of bleomycin as described previously (Akhmetshina et al., 2012). Briefly these are male C57BL6/6 mice (*n* = 4) were repeated injected subcutaneously with 100 μL of 0.5 mg/ml bleomycin every other day for 4 weeks. 0.5% NaCl injection serves as controls. After sacrifice tissue was processed and sections cut. After wax removal sections were blocked with 2% BSA for 1 h antibody to Gremlin-1 was incubated overnight at 1:50 dilution (AB4716, Abnova, Taiwan) or matched isotype control antibody in immunohistochemistry buffer at 4°C and then developed with DAB substrate. Tissue was counterstained with haemotoxylin before dehydration through ascending ethanols and coversliped. Images were taken with a microscope at X20 magnification.

Tissue was also stained by Masson’s trichrome stain as described previously (Chakraborty et al., 2017), this demonstrates connective tissue. Images were taken with a light microscope at X10 magnification.

### Statistics

In many comparisons we used Student’s *t*-test and in multiple comparisons we used Analysis of Variance (ANOVA) to compare with a *P* < 0.05 considered significant.

## Results

### Gremlin-1 Increases Myofibroblast Transition

**FIGURE 1:**
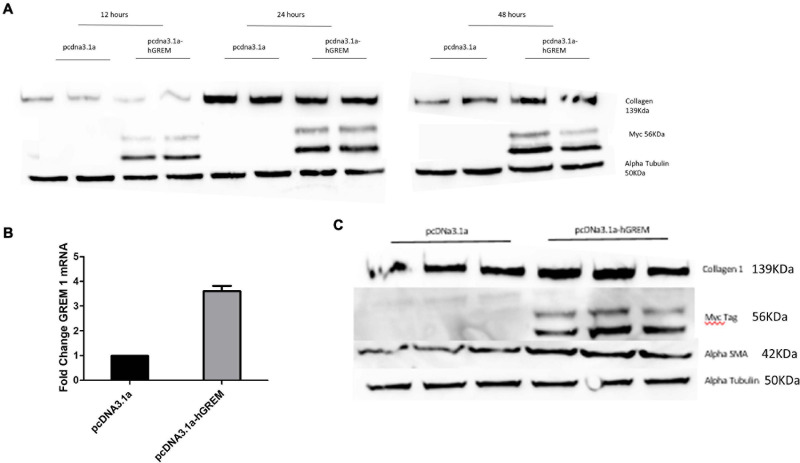
Gremlin-1 increases myofibroblast differentiation. **(A)** Normal human dermal fibroblasts were transfected with pcDNA3.1a construct with or without the human Gremlin-1 gene. Western blots were conducted at 12, 24, and 48 (*n* = 3) h to assess its affects on the key ECM molecule collagen 1 (*n* = 3). **(B)** qPCR was performed at 48 h to assess the transcription of Gremlin-1. Data is normalized to 18s and shown as fold change compared to empty vector plasmid (*n* = 3). **(C)** Gremlin-1 increases myofibroblast marker alpha smooth muscle actin. Normal human dermal fibroblasts were transfected with the plasmid construct pcDNA3.1a with or without the insert for human Gremlin-1 as detailed in the methods. Forty-eight hours post-transfection cells were lysed and a western blot performed with specific antibodies. Alpha Tubulin is the loading control (*n* = 3).

Due to previously demonstrating that Gremlin-1 recombinant protein increased collagen we sought to define this using a different model system in which endogenous Gremlin-1 is used. We used a gremlin-1 overexpression plasmid with a myc-tag for identification as Gremlin-1 antibodies, although commercially available are not specific, with either no signal present or bands at the incorrect molecular weight. Transfection of normal human dermal fibroblasts with an over expressing Gremlin-1 vector increased Collagen 1 protein compared with control vector only particularly after 48 h post transfection (Figure 1A). Gremlin-1 mRNA was also increased in the overexpressed cells (Figure 1B).

**FIGURE 2:**
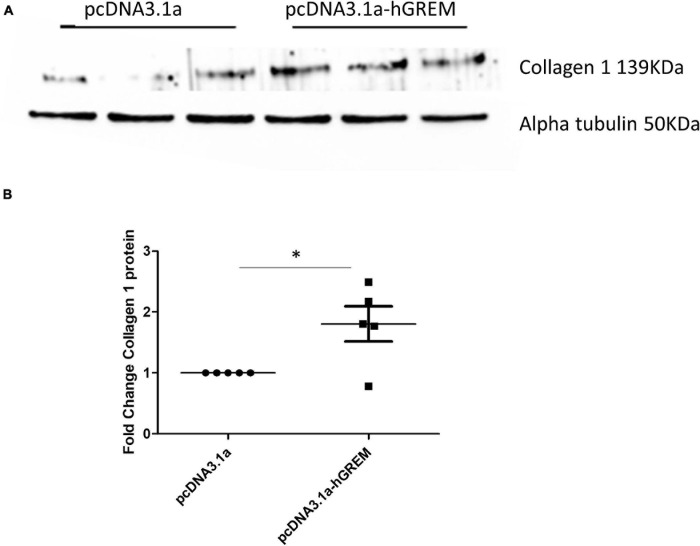
Gremlin-1 overexpression conditioned medium increased collagen expression. **(A)** Normal human dermal fibroblasts were transfected with plasmid pCDNA3.1a with or without the insert for human Gremlin-1, after 48 h the media was removed and then placed onto new dermal fibroblasts after a further 48 h cells were lysed and subjected to western blotting for the specified proteins. **(B)** Densitometric analysis of collagen after plasmid transfection data is normalized to alpha tubulin expression and shown as fold change compared to pcDNA3.1a (*n* = 5). The meaning of ‘*’ is significantly different students *t* test.

xInterestingly, the myofibroblast marker alpha smooth muscle actin (αSMA) was also increased in gremlin-1 overexpressing cells (*n* = 3) (Figure 1C). The transfer of conditioned media from transfected to un-transfected cells yielded similar results, in terms of increased Collagen 1 to that of transfected cells (*n* = 3) (Figures 2A,B). This result would connote gremlin-1 is acting in cytokine-like manner or Gremlin-1 signaling is leading to an increase in pro-fibrotic cytokine(s), such as TGF-β1.

### TGF-β1 Does Not Increase Gremlin-1 mRNA in Dermal Fibroblasts

Previous studies have shown a link between TGF-β1 signaling and Gremlin-1 in both ocular and kidney tissue, with neutralization of TGF-β2 reducing Gremlin-1 expression in mesangial cells (McMahon et al., 2000). It is also clear that in SSc, TGF-β1 is a clear and potent pro-fibrotic molecule (Lafyatis, 2014; Györfi et al., 2018). We thus hypothesized that stimulation with TGF-β1 would increase the transcription of Gremlin-1 mRNA. Here we showed that treatment of Normal Healthy Dermal Fibroblasts (NHDF) with 10 ng/mL recombinant TGF-β1 had no significant effect on Gremlin-1 mRNA expression at both 24 and 48 h, fold change of 0.97 and 1.12, respectively (*n* = 5, Student’s *t*-test 24 h *P* = 0.37 and 48 h *P* = 0.95).

### Gremlin-1 Promotes Migration in Dermal Fibroblasts

**FIGURE 3:**
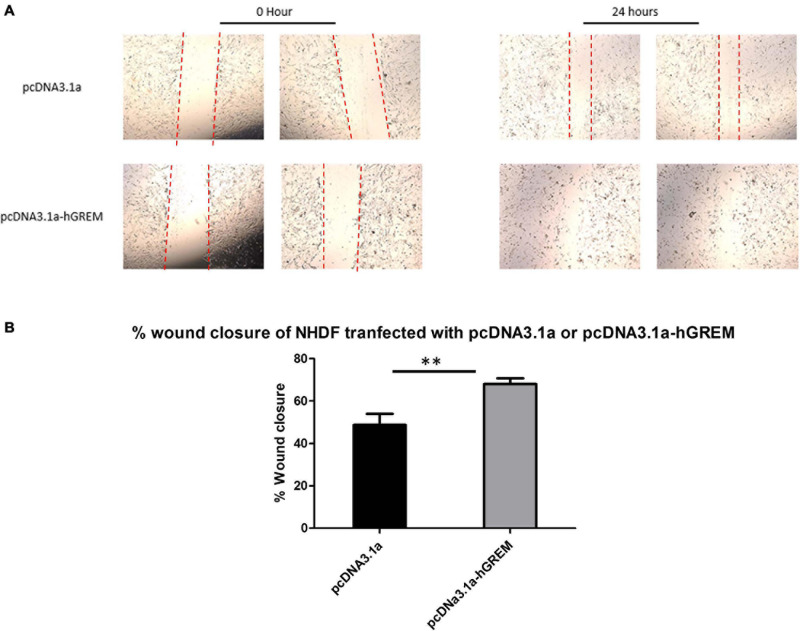
Gremlin-1 increased migration in normal healthy dermal fibroblasts. Normal human dermal fibroblasts were transfected with the plasmid construct pcDNA3.1a with or without the human Gremlin-1 insert. Cells were seeded at high confluency and a scratch assay performed. **(A)** Representative images. **(B)** Images were then quantified in Image J software and wound closure obtained as a percentage of the initial wound. Gremlin-1 increased wound closure in these cells at 24 h time point (*n* = 3, Student’s *t*-test, ^∗∗^*P* ≤ 0.0016).

Standard wound healing scratch assays were conducted to investigate the migratory effects of increased Gremlin-1 on the NHDF. The activation of fibroblasts *in vivo* results in a changed intermediary phenotype resulting in increased migration and proliferation, before their eventual conversion to the prototypical myofibroblast (Schmidt et al., 1995). Figure 3A demonstrates representative image of wound healing after transfection and scratching. Analysis of migration as a percentage of wound closure via image J software revealed increased migration in NHDF overexpressing Gremlin-1 as compared to vector only expressing construct (Figure 3B). Percentage wound closure from the initial wound was 50% in the pcDNA3.1a construct and 68% in the construct containing the Gremlin-1 insert (Figure 3B) (*n* = 3, *t*-test, *P* = 0.0012^∗∗^, Values are mean ± SEM).

### Gremlin-1-Mediated Increase Collagen 1 Can Be Attenuated by siRNA Knockdown

**FIGURE 4:**
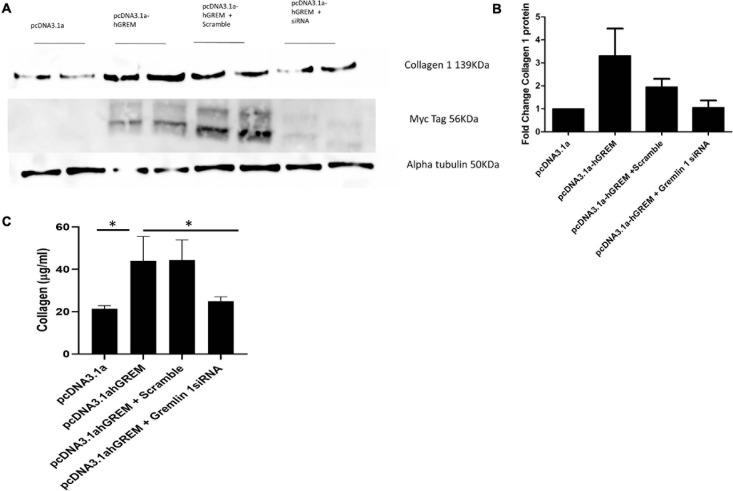
siRNA to Gremlin-1 reduces collagen expression. Treatment of NHDF wit Gremlin-1 siRNA: NHDF were co transfected with the plasmid construct pcDNA3.1a and pcDNA3.1a-hGREM and either a non-targeting (scramble) or Gremlin-1 targeting siRNA. **(A)** Representative western blot and **(B)** densitometric analysis (*n* = 4, *t*-test *P*-value vs. pcDNA3.1a—pcDNA3.1a-hGREM = 0.060653 (*SD* ± 2.85), pcDNA3.1-hGREM + scramble = 0.044446^∗^ (*SD* ± 0.82), pcDNA3.1a-hGREM + Gremlin-1 siRNA (*SD* ± 0.63) = 0.723193 and *P*-value of pcDNA3.1a-hGREM + scramble vs. pcDNA3.1a-hGREM + Gremlin-1 siRNA = 0.0889. **(C)** Collagen was quantified by Sircol assay after transfection of pcDNA3.1, pCDNA3.1a-hGREM, pcDNA3.1a-hGREM and scramble siRNA or pcDNA3.1a-hGREM and siRNA Gremlin-1.

Gremlin-1 transfected NHDF cells were co-transfected with Gremlin-1 siRNA to determine if the effects on the ECM proteins could be attenuated. As the results in Figure 4A show when co-transfected with siRNA Collagen 1 does not increase upon overexpression of Gremlin-1 (Figure 4B). Collagen secretion was also significantly reduced by siRNA to Gremlin-1 also (Figure 4C) (*P* = 0.0095 ANOVA).

This data combined with the data collected from the media transfer assays further suggestive of a rapidly acting cytokine like role for Gremlin-1. Previous data shows Gremlin-1 mRNA is unchanged by rTGF-β1 treatment, we further investigated if the secretion of TGF-β1 was affected by the over expression of Gremlin-1 protein.

### Gremlin-1 Over Expression Causes a Significant Increase in TGF-β1 Secretion and Activation

**FIGURE 5:**
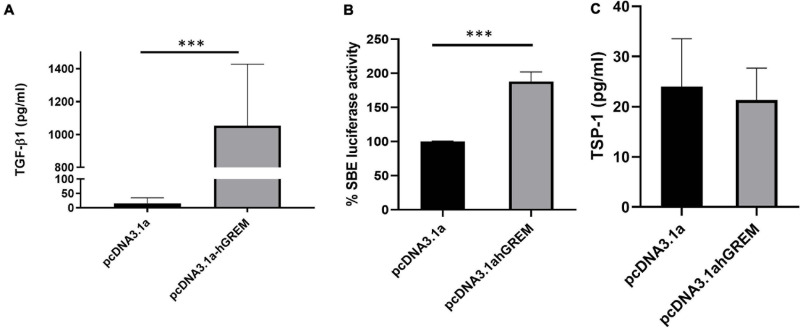
Gremlin-1 increases TGF-β1 protein. **(A)** TGF-β1 was measured by ELISA after overexpression of pcDNA3.1 or pcDNA3.1hGREM, ^∗∗∗^*P* ≤ 0.0001; Student’s *t*-test; *n* = 3). **(B)** Luciferase activity of SBE construct after media was placed onto the cells after transfection with pcDNA3.1 or pcDNA3.1hGREM, *P* ≤ 0.001; Student’s *t*-test; *n* = 3. **(C)** TSP-1 levels were measured after plasmid transfection in the conditioned media. No significant difference, *P* ≥ 0.05; Student’s *t*-test; *n* = 3.

We have demonstrated that Gremlin-1 leads to increased ECM in skin fibroblasts. We further demonstrated that TGF-β1 does not regulate Gremin-1 but queried if Gremlin-1 lead to increased TGF-β1? To test this hypothesis we overexpressed Gremlin-1 in dermal fibroblasts and quantified secreted TGF-β1 by specific ELISA. Overexpression of Gremlin-1 led to increased levels of TGF-β1 secretion compared to vector only transfected cells. In pcDNA3.1a NHDF mean TGF-β1 secretion was 15.2 pg/mL, whilst in the supernatant of NHDF transfected with pcDNA3.1a TGF-β1 secretion was 1,054 pg/mL at 48 h (*n* = 3, Student’s *t*-test, *P* = 0.0001^∗∗∗^, values are mean ± SE) (Figure 5A). Using a luciferase reporter assay we could find activation of a Smad binding element after transfection of cells with vectors and transfer of conditioned medium to transfected cells, with a mean increase in luciferase of over 80% (*P* ≤ 0.001 Student’s *t*-test). This indicates that the TGF-β1 in the medium is active and productive (Figure 5B).

Because we had found upregulation of TGF-β1 and through the Smad reporter assay, activation, we sought to measure the expression of thrombospondin-1 (TSP-1). TSP-1 is a potent activator of TGF-β1 from its latent non-active form and thus elevated TSP-1 would indicate a possible mechanism of activation. After overexpression of Gremlin-1 compared to non-insert plasmid we found no significant difference in TSP-1 levels in the media (Figure 5C). This suggests that TGF-β1 increased through Gremlin-1 is not mediated by TSP-1 increases.

### Inhibitors to TGF-β1 Attenuate the Gremlin-1 Mediated Increase in Collagen 1 Protein

**FIGURE 6:**
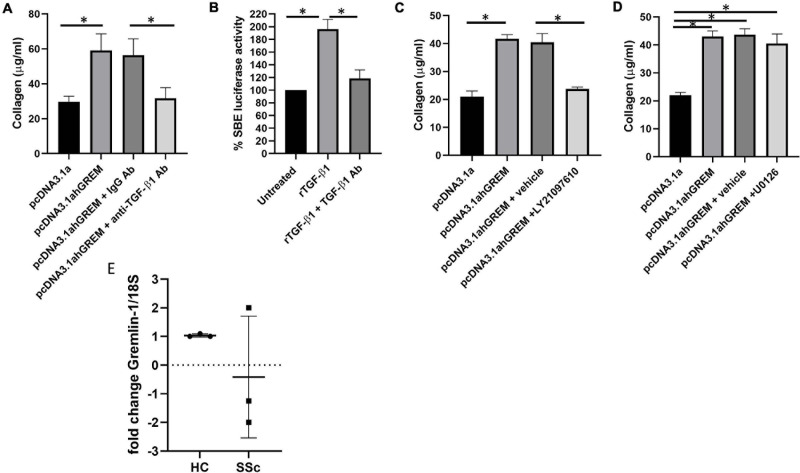
Gremlin mediates its effects through TGF-β/Smad signaling. **(A)** Collagen is reduced by TGF-β1 neutralizing antibody. Cells were treated with isotype matched antibody or TGF-β1 neutralizing antibody and either empty pcDNA3.1a or pcDNA3.1a-hGREM. ^∗^Significantly different *P* = ANOVA pcDNA3.1 vs pcDNA3.1a-hGREM. *P = pcDNA3.1a-hGREM vs. pcDNA3.1a-hGREM and TGF-β1 antibody. **(B)** TGF-β1 neutralizing antibody reduced SBE luciferase in fibroblasts *P* ≤ 0.001 rTGF-β1 vs. rTGF-β1 and neutralizing antibody. **(C)** Collagen is reduced with LY2109761 with gremlin-1 overexpression. **(D)** U0126 ERK (10 μM) inhibitor has no effect of Gremlin-1-induced collagen *P* ≥ 0.05. **(E)** Gremlin-1 was quantified by qPCR in HC and SSc dermal fibroblasts. Data is individual donors. Dashed line horizontal is 0 fold change *n* = 3.

Because we have demonstrated a significant increase in TGF-β1 after Gremlin-1 over expression we sought to determine if inhibitors of TGF-β1 pathway would attenuate the Gremlin-1-mediated increase in collagen expression. We overexpressed Gremlin-1 using our overexpression construct plus an irrelevant IgG control antibody or 1 μg of neutralizing TGF-β1 antibody and measured released collagen. Figure 6A demonstrates a significant increase of collagen release by the Gremlin-1 vector compared to the pcDNA3.1s vector alone, which was retarded by incubation with the TGF-β1 neutralizing antibody but not with the isotype-matched Ig control antibody (*P* = 0.002 ANOVA; *n* = 3). We confirmed neutralizing ability of the antibody by incubating the antibody with recombinant TGF-β1 protein or not and using the SMAD reporter luciferase assay, Figure 6B demonstrates significant reduction of luciferase activity with the anti-TGF-β1 antibody (Figure 6B, *P* ≤ 0.001 ANOVA; *n* = 3).

**FIGURE 7:**
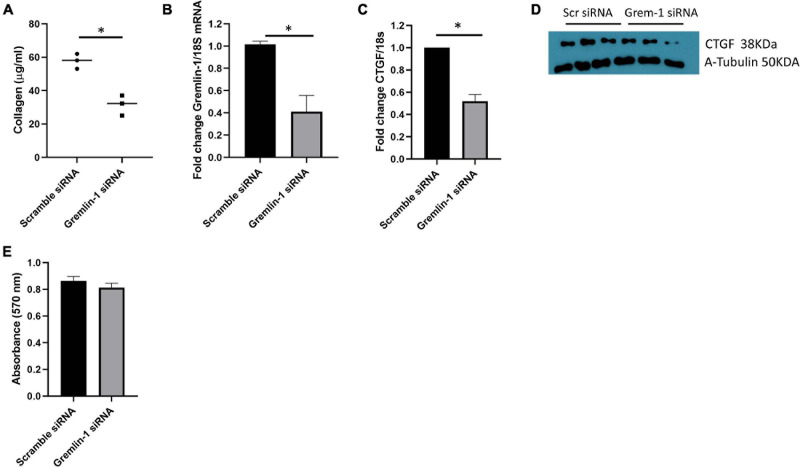
Knockdown of Gremlin-1 in SSc mediates reduced ECM in SSc cells. **(A)** SSc fibroblasts from 3 donors were transfected with scramble or Gremlin-1 siRNA and collagen was quantified by Sircoll assay. Each point is individual donor and the horizontal bar is the mean, *P* = 0.0038; Student’s *t*-test; *n* = 3. **(B)** Confirmation of Gremlin-1 knockdown by qRT-PCR after scramble or Gremlin-1 siRNA, *P* = 0.0022; Student’s *t*-test; *n* = 3. **(C)** CTGF levels after siRNA scramble control or Gremlin-1 siRNA transfection. Data is normalized to 18S and shown as fold change to scramble siRNA controls, *P* = 0.0002; Student’s *t*-test; *n* = 3. **(D)** Western blot of CTGF and α-tubulin to confirm equal protein loading after transfection of scramble or siRNA targeting Gremlin-1 in three different SSc donors. **(E)** Absorbance at 570 nm after siRNA Gremlin-1 or scramble control siRNA. No significant difference, *P* = 0.13; Student’s *t*-test; *n* = 3. The meaning of ‘*’ is significantly different students *t* test.

The TGFβ inhibitor LY2109761 is a dual inhibitor which inhibits both TGFβRI and TGFβRII (Melisi et al., 2008), thereby blocking the TGF-β pathway in cells. Use of this inhibitor attenuated the Gremlin-1-mediated collagen 1 increase in the NHDF Figure 7C demonstrates that incubation with the TGF-β receptor inhibitor LY2109761 reduced collagen release compared to Gremlin-1 overexpression or Gremlin-1 and the vehicle control (*n* = 3) (Figure 6C). The downstream mediator appears to be involved in the signal transduction of Gremlin-1. Although TGF-β1 signals primarily through the Smad signaling pathway other what are termed non-canonical pathway can also play a role. The non-canonical pathway comprises activation of the TGF-β receptor and instead of Smad activates Ras, Raf and the ERK signaling. We therefore used inhibitors to MEK U0126 to block the ERK pathway in fibroblasts plus and minus plasmids for Gremlin-1. Figure 6D demonstrates that inhibition with MEK inhibitor U0126 had no effect on Gremlin-1 mediated increased collagen secretion (*P* ≥ 0.79 pcDNA3.1ahGrem vs. pcDNA3.1ahGrem and U0126; *P* = 0.0137 pcDNA3.1a vs. pcDNA31.ahGrem).

### Gremlin-1 mRNA Is Not Significantly Different in SSc Fibroblasts

Systemic sclerosis is a highly heterogeneous disease and recent theories in the field have shifted toward the ideology that SSc has multiple subtypes, oppose to the classically defined limited and diffuse cutaneous (Hinchcliff and Mahoney, 2019). Gremlin-1 was not increased at the mRNA level in early diffuse SSc patient skin samples (patient *n* = 3). In the SSc skin patient samples 1 patient showed a twofold increase in Gremlin-1 mRNA when compared to healthy control, however the other 2 patients showed a decrease in Gremlin-1 mRNA (−2 and −1.25-folds) (Figure 6E). It is not possible to quantify Gremlin-1 at the protein level due to non-specific antibodies.

### Gremlin-1 Small Interfering RNA Reduced ECM in SSc Dermal Fibroblasts

Using our model system of overexpression of Gremlin-1 we could find elevated ECM markers associated with fibrosis that is associated with enhanced TGF-β signaling. We sought to reduce gremlin expression in 3 SSc early diffuse patients samples. Employing small interfering RNA (siRNA) we can see a reduction in collagen release compared to non-targeting control siRNA at the same concentration 57.8 μg/ml scramble siRNA (*SD* = 4.5) vs. 31.5 μg/ml Gremlin-1 siRNA (*SD* = 6.1), *P* = 0.0038; Student’s *t*-test *n* = 3 donors (Figure 7A). We could confirm successful siRNA mediated knockdown of Gremlin-1 by measuring the mRNA expression after knockdown, compared to scramble siRNA Gremlin-1 siRNA reduced Gremlin-1 expression by over 60% (Figure 7B), *P* = 0.0022; Student’s *t*-test *n* = 3. In light of the effect of overexpression of Gremlin-1 inducing collagen via enhanced TGF-β signaling we sought to examine a downstream target gene of TGF-β1 CTGF (Black and Trackman, 2008) after siRNA knockdown of Gremlin-1. CTGF is a known target of TGF-β1 and mediates fibrosis. Figure 7C demonstrates that CTGF mRNA is reduced by over 50% in Gremlin-1 siRNA knockdown cells compared to scramble (*P* = 0.0002; Student’s *t*-test *n* = 3) and this reduction of CTGF was reduced at the protein level (Figure 7D). To examine if ablation of Gremlin-1 reduced cell proliferation we undertook an MTT assay after siRNA scramble or siRNA to Gremlin-1. No difference was seen in cell proliferation (Figure 7E). This suggests that cell proliferation plays no role in the reduction of collagen.

### IL-6 but Not IL-13 nor IL-11 Increase Gremlin-1 in Dermal Fibroblasts

**FIGURE 8:**
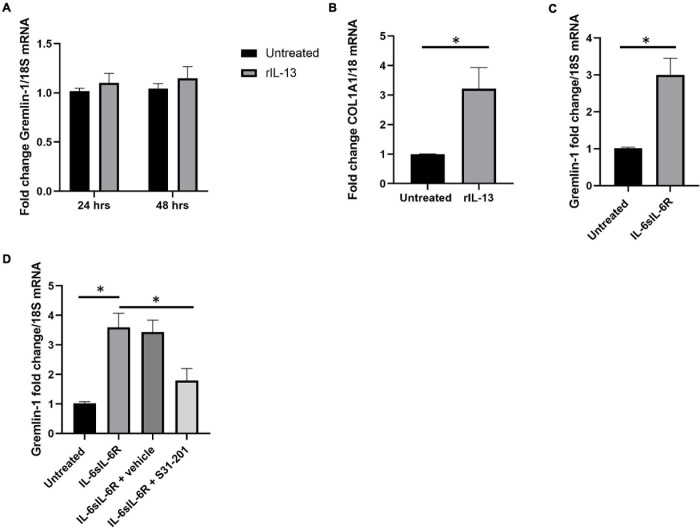
IL-6 but not IL-13 or IL-11 mediates Gremlin-1 upregulation. **(A)** Gremlin-1 expression in healthy dermal fibroblast after stimulation with recombinant IL-13 at 24 and 48 h time points. Data is mean and *SD* and is normalized to 18S expression and shown as fold change to untreated cells. **(B)** COL1A1 gene expression is elevated after IL-13 incubation Data is mean and *SD* and is normalized to 18S Data is the mean and *SD*. **(C)** Gremlin-1 gene expression after IL- trans signaling. Data is the mean and SD and is normalized to 18S and shown as fold change. ^∗^*P* = 0.0015; Student’s *t*-test *n* = 3. **(D)** Gremlin-1 gene expression was quantified by qPCR. Cells were treated with IL-6 and sIL-6 receptor or the STAT3 inhibitor S31-201. Cells were lysed and qPCR performed. Data is the mean and *SD* and is normalized to 18S and set as fold change.

Although TGF-β1 was induced by Gremlin-1, TGF-β1 itself did not induce Gremlin-1, therefore we sought to determine if IL-13 induced Gremlin-1 as IL-13 is a known pro-fibrotic molecule particularly elevated in SSc (O’Reilly, 2013). Incubation of IL-13 at both 24 and 48 h did not lead to a significant increase in Gremlin-1 expression in healthy dermal fibroblasts (Figure 8A, no significant difference, *P* ≥ 0.05 Two way ANOVA; *n* = 4).

To confirm that the recombinant IL-13 we used was indeed functional we measured colagen1A1 expression by qPCR (Figure 8B), there was a significant 3.2-fold increase in COL1A1 gene expression at 24 h (*SD* = 0.72), *P* = 0.0009; Student’s *t*-test; *n* = 4 (Figure 8B). This indicates that the IL-13 is functional and does not modify Gremlin-1. We had previously described a role for IL-6 trans signaling in Gremlin-1 and fibrosis (O’Reilly et al., 2014), we confirmed here that incubation with both IL-6 and soluble IL-6 Receptor leads to upregulation of Gremlin-1 (Figure 8C), *P* = 0.0015; Student’s *t*-test. IL-6 signals through the IL-6 Receptor but this is not present on the surface of dermal fibroblasts and needs to be added *in vitro* to signal through what is coined trans signaling (O’Reilly et al., 2014). The main downstream signaling mechanism from IL-6 signaling is through Janus Kinases (JAKs) and subsequent phosphorylation of STAT3 leading to gene expression. To corroborate the role of STAT3 in IL-6 mediated Gremlin-1 induction we incubated dermal fibroblasts with IL-6/IL-6R combination and the STAT3 inhibitor S31-201 which resulted in diminished induction of Gremlin-1 (Figure 8D). A previous study *in vivo* also showed induction of STAT3-mediated Gremlin-1 in lung fibrosis (Wong et al., 2014) and a recent paper demonstrated that IL-6 trans signaling in idiopathic pulmonary fibroblasts elevated Gremlin-1, which was reduced with tocilizumab (Epstein Shochet et al., 2020).

Because IL-11 is an IL-6 family cytokine, that uses similar downstream signaling pathways as IL-6 and we have shown that this is both pro-fibrotic in SSc and is also elevated in the sera of patients (Adami et al., 2021) we posited that this cytokine would increase Gremlin-1 levels in dermal fibroblasts. Using the dose that we have found to increase myofibroblast generation (10 ng/ml) we examined Gremlin-1 expression after 24 h stimulation and found no significant difference in expression compared to untreated cultures (untreated 1 vs. 1.15-fold change *P* ≥ 0.005; *n* = 3).

### Gremlin-1 Is Elevated in Bleomycin Model of Fibrosis

**FIGURE 9:**
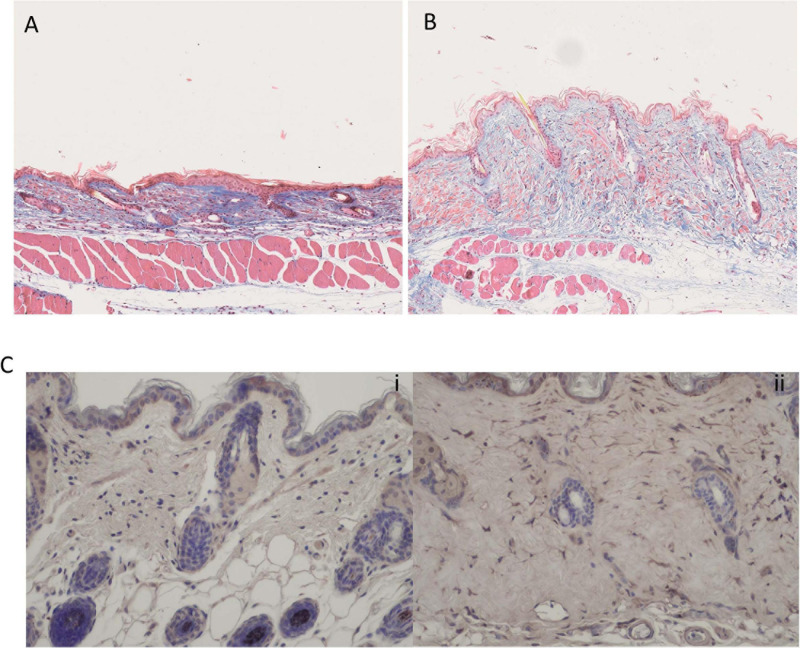
Bleomycin treated mice have elevated skin fibrosis and Gremlin-1 expression. **(A)** Representative Trichrome staining of NaCl treated mice. **(B)** Representative Trichrome staining of bleomycin treated mice- showing excessive skin thickening. Magnification ×10. **(C)** Immunohistochemistry of Gremlin-1 using specific antibody to Gremlin-1 (i) NaCl treated mice and (ii) bleomycin treated mice. Hematoxylin is the nuclear stain. Magnification ×20.

Finally, we used the bleomycin model of skin fibrosis, which is a classic model of skin fibrosis that is dependent on inflammation and examined using immunohistochemistry Gremlin-1 expression. Figure 9A shows skin thickness in NaCl injected control mice and Figure 9B shows significantly different thickness in bleomycin treated mice. Figure 9Ci demonstrates few cells expressed Gremlin-1 in the control NaCl-treated mice whereas the bleomycin-treated mice the dermal fibroblasts expressed Gremlin-1 (Figure 9Cii).

## Discussion

Gremlin-1 has been previously found to be elevated in SSc tissues and has been associated with various tissue fibrosis states including the liver, lung, eye, and kidney (Koli et al., 2006; Lee et al., 2007; Yang et al., 2012; Wellbrock et al., 2014). Building upon our previous studies we sought to examine the role of Gremlin-1 in skin fibrosis further. Rather than using recombinant protein to elicit the effects we utilized an overexpression system in primary dermal fibroblasts.

In this study we demonstrated that Gremlin-1 increases collagen 1 at the protein level in dermal fibroblasts and functional enhancement of the wound healing/fibrosis response. Alongside increased collagen 1 protein the study also found an increase the secretion of TGF-β1 and is in line with data published in renal cells (Rodrigues-Diez et al., 2012). Given that TGF-β1 is a major pro-fibrotic cytokine both in liver and lung fibrosis but also in SSc (Wei et al., 2010; Lafyatis, 2014; Frangogiannis, 2020), we hypothesized that this may lie upstream of Gremlin-1 regulation and thus predicted that TGF-β1 stimulation would lead to elevated Gremlin-1.

Interestingly, treatment of the fibroblasts with recombinant human TGF-β1 did not increase Gremlin-1 expression. Commercially, available antibodies to Gremlin-1 are notoriously unreliable and in our hands, we were unable to gain reliable and reproducible results, hence the measurement of Gremlin-1 at mRNA level and not the protein level. So, we cannot rule out the possibility that TGF-β1 does regulate Gremlin-1 post transcriptionally.

Media transfer experiment conducted in this study support the hypothesis that Gremlin-1 is either acting as a cytokine itself or increasing secretion of profibrotic cytokine(s) into the extracellular space. This would further support the hypothesis that Gremlin-1, perhaps via its downstream affects, is shifting the quiescent cell to a myofibroblast phenotype; a key phenotype in skin fibrosis (Antic et al., 2013).

We could not find evidence that TGF-β1 upregulates Gremlin-1 expression at multiple times points but the opposite appears true in that Gremlin-1 overexpression led to significantly elevated expression of TGF-β1 into the cell media. This was further confirmed to be activated as this media could activate the Smad reporter system, which is a Smad-responsive element linked to luciferase, further supporting activation of TGF-β1 by Gremlin-1. Because one of the most potent activators of TGF-β1 is TSP-1 (Frangogiannis, 2020) in the matched media we examined if this was elevated. TSP-1 was not elevated by Gremlin-1 suggesting that activation of TGF-β1 is independent of TSP-1. Other activators of TGF-β1 include Reactive Oxygen species and the integrin ανβ6 (Munger et al., 1999). Indeed gremlin-1 has been shown to alter the expression of β3 integrin to stabilize the VEGF receptor. Thus, it could be possible, but requires confirmation, that Gremlin-1 activates TGF-β1 leading to increased fibrosis through increased integrin enhancement.

Because we saw upregulation of TGF-β1 and that we know TGF-β1 is a potent pro-fibrotic molecule leading to fibrosis in multiple organs and cells (Frangogiannis, 2020) we postulated that Gremlin-1-mediated fibrosis is through TGF-β1 signaling causing a fibrotic loop sustaining fibrosis. Thus, we used neutralizing antibodies to TGF-β1 to neutralize this and compared to isotype matched antibody we could see impressive reductions in collagen. We confirmed suppression of TGF-β1 signaling through the luciferase reporter assay and recombinant TGF-β1. To confirm that this was receptor-mediated we used the classic TGFβ receptor inhibitor and found that this also reduced Gremlin-1-mediated collagen expression. Because TGF-β1 can activated the non-canonical pathway and primarily MAPK we used the potent MAPK inhibitor and found that this did not diminish the increase in collagen mediated by Gremlin-1. We thus confirm that Gremlin-1 mediated fibrosis primarily through TGF-β1 canonical pathways, however, other non-canonical pathways could be involved as we cannot exclude the p38 pathway and others. Studies by Sethi et al. (2013) demonstrated that Gremlin-1 utilized the canonical Smad and MAPK p38 pathway to induce ECM protein and Lysyl oxidase (LOX) genes in trabecular meshwork cells. Our work demonstrates that only the canonical pathway is playing a role in ECM deposition in these cells and the difference is likely due to different cells types: meshwork cells of the eye have a more endothelial phenotype.

A limiting factor in this study is the patient number, in our limited sample Gremlin-1 did not appear increased; however, SSc is an extremely rare and heterogeneous disease and securing patient samples is extremely difficult. However using the patient samples we had we used siRNA to Gremlin-1 we could significantly reduce Gremlin-1 and there was a corresponding significant decrease in collagen and the target gene of TGF-β1 CTGF was also significantly supressed at both the mRNA and protein level, suggesting that this is reducing TGF-β signaling. It is possible also that local concentrations of Gremlin-1 in SSc are elevated in and around the skin affected or indeed in the serum. A commercial ELISA does exist for Gremlin-1 but we are yet to be convinced of its specificity. Significantly elevated levels of Gremlin-1 has been demonstrated in renal fibroblasts and in idiopathic pulmonary fibrosis (Walsh et al., 2018). It could be that the mRNA expression is unchanged in the SSc samples but the protein level is much increased.

We next sought to examine the role of an inducer of Gremlin-1 in these cells. The primary reason IL-13 was chosen is that this is a highly fibrotic cytokine which is significantly elevated in SSc (O’Reilly, 2013). Incubation of dermal fibroblasts with recombinant IL-13 did not lead to elevated Gremlin-1. We interpret this that IL-13 does not directly lead to Gremlin-1 expression. We could confirm it was functional as collagen mRNA expression increased as expected. IL-6 trans signaling, which is signaling using the soluble IL-6 receptor and IL-6 was found by us to induce collagen (O’Reilly et al., 2014) and we extended this further by demonstrating that IL-6-mediated-Gremlin-1 is facilitated through STAT3-as the STAT3 inhibitor S31-201 significantly retarded this effect. This is in keeping with our previous work (O’Reilly et al., 2014) and also recent work in lung fibrosis and most recently bone marrow-derived stromal cells were found to upregulate Gremlin-1 via IL-6 trans signaling and this could be retarded with the use of an IL-6 monoclonal antibody (Clark et al., 2020). Given the importance of IL-6 in SSc (O’Reilly et al., 2012, 2013), with significantly elevated levels in the blood and tissues (Hasegawa et al., 1999), a downstream target of IL-6 such as Gremlin-1 may be a more realistic target in this disease as the importance of IL-6 in normal immune functions is critical. Analysis of the Gremlin-1 promoter sequence demonstrates that it has STAT binding sequenced contained within. The relevance of inflammatory regulation of Gremlin-1 is demonstrated in our recent study in which RNA sequencing of skin fibroblasts from mice with a deletion in the nfkb subunit c-Rel revealed significantly reduced levels of Gremlin-1 in KO compared to wildtype mice (Worrell et al., 2020). Because IL-11 is an IL-6 family cytokine and we have recently described its pro-fibrotic role in SSc (Adami et al., 2021), we hypothesized that this would lead to increased Gremlin-1 expression similar to IL-6 stimulation. Surprisingly, we found no increased expression of Gremlin-1 after stimulation, suggesting some specificity to its response. This could be attributed to the fact that IL-11 stimulation in stromal cells preferentially chooses ERK signaling over STAT3 signaling (Adami et al., 2021).

Finally, we utilized the bleomycin animal model of fibrosis and determined Gremlin-1 expression *in situ* and found, compared to the control NaCl mice, that Gremlin-1 expression in fibroblasts was elevated. This further underscores the importance of Gremlin-1 in dermal fibrosis and speculate that this is inflammation-driven.

Overall, this study is demonstrating a role for Gremlin-1 in the accumulation of ECM molecules in SSc, which could be used a novel target to not only investigate treatments into the disease but further assess its etiology. Small molecule inhibitors to Gremlin-1 are not available yet but the target would be a valid one as post-natally expression of Gremlin-1 is negligible. Furthermore, activation of Gremlin-1 maybe a common pathway of all fibrotic diseases, which is a component of 45% of all deaths in the western world (Wynn, 2008).

## Data Availability Statement

The original contributions presented in the study are included in the article/supplementary material, further inquiries can be directed to the corresponding author/s.

## Ethics Statement

The studies involving human participants were reviewed and approved by the South Tyneside REC, United Kingdom. The patients/participants provided their written informed consent to participate in this study. The animal study was reviewed and approved by the Erlangen.

## Author Contributions

LD: methodology and data gathering and analysis. JH, MB, LS, and JD: data gathering and analysis. NF: reagents. SP: provided reagents and writing. RS: conceptualization and reagents. SO’R: conceptualization, project management of whole project, supervision, and primary writing of manuscript. All authors contributed to the article and approved the submitted version.

## Conflict of Interest

The authors declare that the research was conducted in the absence of any commercial or financial relationships that could be construed as a potential conflict of interest.
